# 

**Published:** 1847-02

**Authors:** 


ARTICLE XVII.

NATIONAL MEDICAL CONVENTION.

To Subscribers.—The low price of the Journal makes it necessary that we insist upon prompt payments. We hope our subscribers will understand this without further comment and at once forward the amount of the subscription. Those who have not paid for the present volume will please forward for it and the ensuing year, as our terms are *payment in advance*. Payment may be made to either of the editors or to our authorized agents, and forwarded post-paid by mail at our risk.

### RUSH MEDICAL COLLEGE.

### TO READERS AND CORRESPONDENTS.

We have received communications from Dr. P. Henry Clark and Prof. David Prince.

The following works have been received:—

Adulteration of various Substances used in Medicine and the Arts, with the Means of Detecting them: intended as a Manual for the Physician, the Apothecary and the Artisan. By Lewis C. Beck, M.D., Prof. of Chemistry in Rutger's College: Honorary Member of the Medical Society of the State of New York, etc. New York: Samuel S. &William Wood, No. 261 Pearl Street. 1846. pp 333. 12mo.

An Anatomical description of the Diseases of the Organs of Circulation and Respiration. By Charles Edward Hasse, Professor of Pathology and Clinical Medicine in the University of Zurich, etc. etc. Translated and edited by E. W. Swayne, M.D., Physician Extraordinary to H. R. H. the Dutchess of Kent. Philadelphia: Lee & Blanchard. 1846. pp. 277. 8vo. (From the Publishers.)

Latham on diseases of the Heart. Forming the Oct. No. of Bell's Select Medical Library.

Chemistry of the Four Seasons—Spring, Summer, Autumn, and Winter: An Essay principally concerning Natural Phenomena, admitting interpretation by Chemical Science, and illustrating passages of Scripture. By Thomas Griffiths, Professor of Chemistry in the Medical College of St. Bartholomew's Hospital, &c. Philadelphia: Lea & Blanchard.—1846. pp. 451. 12mo. From the Publishers. (For sale by Brautigam & Keen, Chicago.)

Summary of the Transactions of the College of Physicians and Surgeons of Philadelphia—from Sept. to Nov. 1846, inclusive.

Lecture on Obstetrics, &c., by G. S. Bedford, M.D., Prof. in the University of New York—Session 1846-7.

An Introductory Lecture, delivered before the class of the Baltimore College of Dental Surgery, by A. Wescott, A.M., M.D., Prof.

Annual Catalogue of the Med. Department of the University of Louisville.

Observations on Intermittent Fever, by William M. Bowling, M.D., of Montgomery, Alabama.

Valedictory Lecture, delivered before the Class in Rush Medical College, by John McLean, M. D. and Professor.

Annual Catalogue of Western Reserve College.

Annual Announcement of the Medical Institute, Cincinatti, Ohio.

Several interesting extracts relative to the use of the etherial vapor have been crowded out.

The following have been received in exchange:—

The Annalist, a Record of Practical Medicine.

The Buffalo Journal and Medical Review.

Stockton & Co's Dental Intelligencer. (Phila.)

Boston Medical and Surgical Journal.

New York Medical and Surgical Reporter.

The Medical Examiner.

The Missouri Medical and Surgical Journal.

The Medical News and Library.

The New York Journal of Medicine.

The Practical Educator and Journal of Health. (Boston.)

The Bulletin of Medical Science.

The American Journal of Insanity.

The Western Journal of Medicine and Surgery.

The Prairie Farmer.

---

### CONTRIBUTORS TO THE ILLINOIS & INDIANA MED. & SURG. JOURNAL.

A. H. Howland, M.D., Ottawa, Ills.
A. G. Henry, M. D., Pekin, Ills.
Ewd. Lewis, M. D., Jackson, Mich.
John McLean, M. D., Jackson, "
Daniel Stahl, M. D. Quincy, Ill.
Edward Mead, M. D., Geneva, Ill.
E. Fisher, Waynesville, Ohio.
H. S. Huber, M. D., Chicago, Ill.
Saml. G. Armor, M.D., Rockford, Ill.
Ira C. Bachus, M.D., Jackson, Mich.
J. G. Cornell, M.D., Spring Arbor, "
G. N. Fitch, M.D., Logansport, Ind.
J. F. Henry, M.D., Burlington, Iowa.
E. Deming, M.D., Lafayette, Ind.
S. B. Thayer, Battle Creek, Mich.

### INDEX.

Abstinence from drinks in the treatment of some diseases, 83
A defence of the medical profession of the U. States, notice of 47
Adulteration of various medicinal substances, by L. C. Beck, M. D., notice of 427
" " " review of 512
Ambrosia Trifida as a remedy for mercurial salivation, 465
Amputation for scrofulous diseases of the joints, by D. Brainard, M. D., Prof. &c. 106
Amputation of the superior maxillary, malar and palate bones, for disease of the antrum: recovery, by Prof. Brainard, 205
Anœmic murmurs, 379
Annalist, new periodical, notice of 427
Appointment of Dr. W. B. Herrick to the medical staff, 266
Apthœ, treatment of, by sulphuric acid, 383
Arsenic, on the use of calcined magnesia as an antidote to, by Prof. Peter, 356
Association of medical superintendents of American institutions for the insane, report of 360
Auscultation, a clinical introduction to the practice of, by H. M. Hughes, M. D., notice of 249

Baldness and falling out of the hair, treatment of 82
Ballard's elements of materia medica and therapeutics, notice of 243, 434
Beck's manual of adulterations of medicine, notice of 427
" review of 512
Bile, in the animal fluid, method of detecting the presence of a minute quantity of 74
Bile, mode of detecting morbid 75
Bismuth, subnitrate of, in Gastralgia, 479
Blindness caused by the use of sulphate of quinia, by Professor McLean, 385
Blood in dropsies, with albuminous urine, an abstract, 158
Blood, physiology and pathology of, report 56
Brodie's clinical lectures on surgery, notice of 245
Budd on diseases of the liver, review, 209
Buffy blood, mode of diagnosing 374

Cancer of the testicle, by John Cooke, M. D., of Vermont, 391
Catalepsy relieved by music, case of 179
Certificates of medical men to quack remedies, 382
Chemical testimony speculative, cases showing with what caution it ought to be received, 478
Chemistry, Hoblyn's, notice of 245
Chemistry of the four seasons, by T. Griffiths, Prof., notice of 542
Closure of external orifice of vaginal canal, operation for, by A. E. Ames, M.D. Roscoe, Ill. 326
Clymer on Fevers, review, 328
Coley on diseases of children, notice of 341
" " " " review 395
Colic, lead, treatment of 365
Convention, national medical 49, 149
Convention, common school, 342, 552
Corpus, luteum, note on the vitellary nature of 568
Copaiba, water of, injections, 375
Cornea, ulceration of, the danger of using certain washes in 81
Congestive Fever, practical remarks on 459
Croup, observations on 452

Death, supposed 84
Death from innoculation with matter from an ulcer succeding the vaccine vesicle, 93
Defence of the medical profession of the United States, notice of 47
Deformities, lectures on the nature and treatment of, by R. W. Tamplin, F. R. C. S. E. review, 132
Delirium tremens, an essay on, by Lieut.EdwinRamsay Long, M.D., U. S. Army, 97
Delivery during sleep, 182
Delivery, rapid, without previous pains, 182
Diabetes mellitus, 83
Diarrhœa of infants, clinical lectures on, Hospital Neckar, 280

Diarrhœa, cases of, with emaciation, coming on after weaning, treated successfully with kreosote, 458
Digestion, a lecture on the physiology of, by Martyn Paine, A.M., M.D., &c., notice of, 145
Diseases of children, a practical treatise on, by J. M. Coley, M.D., notice of, 341
" " " review 395
Diseases of the liver, by Geo. Budd, M. D., review, 209
Diseases of the eye, by S. Littell, M.D., notice of, 243
Dissector, United States, by Prof. Horner, notice of, 341
Dislocation of the elbow, reduction, by Prof. Brainard, 500
Dispensary of Rush Medical College, 549
Dowler on yellow fever, notice of 428
Dr. Herrick, letter from 440
Dropsy, congenital, 459

Eberle's Treatise on the practice of medicine, notice of 147
Editorial notices, 49, 148, 252, 342, 436, 544
Electrical girl, 180
Elements of mat. med. and therapeutics, by Ballard, notice of 243
" " " " review, 434
Emetics in children, 468
Euonymus Atropurpureus, or Wahoo, an essay on the therapeutic virtues of, by E. Andrew, M. D. 15
Epidemic Erysipelas, as it occurred in Logansport, Ia., and its vicinity, by Prof. Fitch, 1
Ergot of rye in lingering labor, conditions for its safe employment, 181
Eruptions on the head and face in children, 350
Etherial vapor, inhalation of, for the prevention of pain during surgical operations, 544
Evans on insanity, 289
Exchanges, our 346

Fascination of serpents, 187
Fevers, distinctive characters of remittent, typhoid and typhus 85
Fever, bilious, the remote and proximate cause of, by S. G. Armor, M.D., Rockford, Ills. 189
Fevers, five dissertations on, notice of, 244
Fevers, diagnosis, pathology, and treatment of, by Meredith Clymer, M.D., review, 328
Fevers and inflammations, on the treatment of, without mercury, by A. G. Henry, M.D., Pekin, Ills. 489
Fordyce on fever, notice of 244
Foreign body in the tongue for thirty two years, 83
Foreign bodies in the organs and tissues, by Prof. Herrick, 115
Floodings, 366

Galvanism, the use of, in lumbago, sprains, &c., 373
Gastralgia, subnitrate of bismuth in 479
Goodhue's address before the Rock River Medical Society, 260
Gonorrhœa, treatment of 375

Hasse on diseases of circulation and respiration, review, 526
Hernia, strangulated case of, by Dr. J. Harvey, 388
Herrick, Dr., letter from 507
Hoblyn's Chemistry, notice of 245
Homeopathy, allopathy & young physic, by J. Forbes, M. D., &c., review, 23
Homeopathy, 456
Hemorrhage, on the mode of arresting 383
Hospitals for the insane, 438
Hospital Neckar, clinical lecture of 286
Hughes on Auscultation, notice of 249
Hydrocephalus, a case of, successfully treated with iodide of potassium, by L. Brackett, M. D., of Rochester, Ind. 393

Injections, cold astringent, in uterine hemorrhage, 91
Insanity, neglect of the study of, by physicians, 174
Infants, milk a cause of disease in 76
Intermittent fever, on the curative medication of 270
Infants, clinical lectures on diarrhœa of 280
Issues of nitric acid in chronic diseases, by P. A. Allaire, M.D. 502
Intermittent fever, combination of carbonate of iron with sulph. quinine in 383
Insanity, observations on, by Prof. Evans, 289

Johnston and Martin, on tropical climates, notice of 244
Journal, notice of our 49, 148
Journal, abstract of articles in 553

Kermes Mineral, on the use of, in diseases of the respiratory organs, 377
Kidney, minute anatomy of, abstract, 155

Lancette Canadienne, notice of 551
Latham on diseases of the heart, notice of 542
Lead colic, treatment of 365
Liston and Mutter's surgery, notice of 250
Littell on diseases of the eye, notice of 243
Liver, diseases of, by Geo. Budd, M. D., &c., review, 209
Lobelia Inflata, on the use of 375
Lunatic Asylums, 148

Mad-dog, case of the bite of, and treatment, by D. Stahl, 118
Magnesia, calcined, as an antidote to arsenic, 356
Magnetism, abstract of researches on 561
Mal-practice, prosecution for, arbitration, 262
Mal-practice in midwifery, 473
Mammary glands, case of premature development of 458
Manual of the diseases of the eye, notice of 243
Materia medica and therapeutics, by E. Ballard, M.D., &c. 243, 434
Medical school, notice of another 149
Medical convention, national, notice of 49, 149
Medical convention at Rockford, notice of 153
Medical science and art, fragments of, by H. J. Bigelow, M. D., review, 35
Medical school, new 442
Medical school in Michigan, 442
Medical institution, Willoughby 443
Medical society, Rock River, preamble and constitution of 252
Medical college, another, in Phila. 53
Medical eras, 573
Medical Books, 550
Mental excitement allayed by music, 207
Mental cultivation and excitement, by A. Brigham, M.D., review, 121
Menstruation in an infant, 189
Mercurial salivation, remedy for 465
Milk, pathological conditions of, as a cause of disease in infants, 76
Miller's surgery, notice of 248
Murphy on Parturition, notice of 427

Non-mercurial treatment of syphilis, 475
Nostrum certifiers, 188

Obstetriciana, by Dr. Dowler, 182
Obituary, Lieut. E. R. Long, M. D., U. S. Army, 54
Operation for closure of vagina, by A. E. Ames, M. D., 326

Paralysis, 68
Phlebitis, 166
Poisons, lecture on antidotes to, abstract, 370
Parturition, lectures on, by E. W. Murphy, M.D., 427
Periodical, new, the annalist, notice of 427
Placenta previa, case of, by W. Butterfield, M.D., 390
Placenta, extraction of, in unavoidable uterine hemorrhage, 92
Polypi of the female urethra, 272
Practice of medicine, by Eberle, notice of, 147
Pregnancy, vaginal, case of 189
Prescriber's pharmacopœia, 542
Premature Interments, 190
Pregnancy, new sign of 375
Purpura hemorrhagica, turpentine in 81

Quackery in New York, 284
Quinine, sulph. blindness from the use of 385
Quinine, use of, by A. W. Benton, M.D., 497
Quiniæ, sulph. new mode of administering 367
Quiniæ, sulph. the endermic application of 368
Quinine, the manner of using, abstract 444
Quiniæ, sulph. in scarlet fever & measles 193

Readers and correspondents, to 95, 191, 287, 384, 480, 575
Rush Medical College 153, 436, 575

Scrofulous diseases of joints, amputations for, by Prof. Brainard, 106
Scabies, on the treatment of 85
Schools as they are and as they should be, 567
Secret remedies, 440
Singultus, 383
Statistics of medical schools, 345
Subscribers, to 552

Materni Naevi 398
[illegible]

Suggestions, useful 367
Surgery, by Liston and Mütter, notice of 250
Surgery, orthopœdic, in Europe, 275
Surgery, by Velpeau, notice of 47
Surgery, Brodie's lectures on, notice of 245
Surgery, Miller's practice of, notice of 248
Syphilis, non-mercurial treatment of 475
Tamplin on deformities, review, 132
Tetanus, case of idiopathic 493
Transactions of medical society of New York, 146
Trismus Nascentium, 347
Tubercular deposite in the cerebellum, 324
Tumor at orifice of meatus urinarius, 176
Turpentine in purpura hemorrhagica, 81

Uterine hemorrhage, cold astringent injections in 91
Uterine hemorrhage, unavoidable, Lee's objections to extracting placenta in 92

Vaccine aphorisms, 93
Vaginal pregnancy, case of 189
Velpeau's surgery, notice of 47
Vision, case of uncommon acuteness of 459

Yellow fev., Dowler on, notice of 428

